# Blind spots in telemedicine: a qualitative study of staff workarounds to resolve gaps in diabetes management

**DOI:** 10.1186/s12913-018-3427-9

**Published:** 2018-08-07

**Authors:** Kathryn Bouskill, Carolyn Smith-Morris, George Bresnick, Jorge Cuadros, Elin Rønby Pedersen

**Affiliations:** 1 RAND Corporation, 1776 Main Street, Santa Monica, CA 90407 USA; 2 Department of Anthropology, Southern Methodist University, Dallas, TX USA; 3 Department of Optometry and Vision Science, University of California, Berkeley, California USA; 4 Google Research, Google LLC, Mountain View, California USA

**Keywords:** Diabetes care, Telemedicine, Diabetic retinopathy, Workarounds

## Abstract

**Background:**

Novel telemedicine platforms have expanded access to critical retinal screening into primary care settings. This increased access has contributed to improved retinal screening uptake for diabetic patients, particularly those treated in Federally Qualified Health Centers (‘safety net’ clinics). The aim of this study was to understand how the implementation of telemedical screening for diabetic retinopathy within primary care settings is improving the delivery of critical preventative services, while also introducing changes into clinic workflows and creating additional tasks and responsibilities within resource-constrained clinics.

**Methods:**

A qualitative approach was employed to track workflows and perspectives from a range of medical personnel involved in the telemedicine platform for diabetic retinopathy screening and subsequent follow-up treatment. Data were collected through semi-structured interviews and participant observation at three geographically-dispersed Federally Qualified Health Centers in California. Qualitative analysis was performed using standard thematic analytic approaches within a qualitative data analysis software program.

**Results:**

The introduction of telemedicine platforms, such as diabetic retinopathy screening, into primary care settings is creating additional strain on medical personnel across the diabetes eye care management spectrum. Central issues are related to scheduling patients, issuing referrals for follow-up care and treatment, and challenges to improving adherence to treatment and diabetes management. These issues are overcome in many cases through workarounds, or when medical staff work outside of their job descriptions, purview, and permission to move patients through the diabetes management continuum.

**Conclusions:**

This study demonstrates how the implementation of a novel telemedical platform for diabetic retinopathy screening contributes to the phenomenon of workarounds that account for additional tasks and patient volume. These workarounds should not be considered a sustainable model of health care delivery, but rather as an initial step to understanding where issues are and how clinics can adapt to the inclusion of telemedicine and ultimately increase access to care. The presence of workarounds suggests that as telemedicine is expanded, adequate resources, as well as collaborative, cross-sectoral co-design of new workflows must be simultaneously provided. Systematic bolstering of resources would contribute to more consistent success of telemedicine screening platforms and improved treatment and prevention of disease-related complications.

**Electronic supplementary material:**

The online version of this article (10.1186/s12913-018-3427-9) contains supplementary material, which is available to authorized users.

## Background

The rise of telemedical services to provide health care in non-traditional settings has been a significant factor in increasing access to specialty care. This has particular benefits for patients in rural and isolated communities, as well as socially disadvantaged patients who are less able to readily access specialty care. While telemedicine solutions for screening, diagnostics, and treatment have been implemented in many clinical settings, such as emergency rooms [1], dermatology [2], psychiatry [3], and pediatrics [4], the focus of this study is on screening for retinal eye diseases that arise as a result of complications from advanced diabetes, or diabetic retinopathy screening (DR screening). Regular diagnostic screening for vision-threatening diabetic retinopathy (VTDR) is crucial for proper diabetes care management. If left untreated, VTDR can lead to permanent vision loss.

Despite the fact that timely detection and treatment of VTDR can be highly effective at preventing vision loss, VTDR persists as the main cause of blindness among working-age adults [5–7]. This is an issue of access both to screening and to subsequent treatment. Telemedicine platforms have filled many of the gaps in access to DR screening, offering a cost-effective way to detect preventable disease through primary care settings [8–10]. Considering the burgeoning population of people with diabetes, addressing the gaps that exist between retinal screening and diabetic retinopathy treatment is crucial.

However, improvements in retinal screening do not consistently lead to the prevention of blindness from VTDR. In some settings, as high as 80% of patients found to have VTDR through retinal screening will not complete follow-up ophthalmological evaluation and treatment recommendations [11]. Although treatments for newly screened patients may exist, these options are not necessarily accessible, feasible, or understood by patients. The complex medical needs of patients with advanced diabetes is exacerbated by structural, economic, and political barriers faced by these patients [12]. To better understand utilization gaps that relate to failure to exercise treatment options, we looked at patients who were found to have VTDR through screening in Federally Qualified Health Centers (which largely serve as ‘safety net’ clinics, and are often strained for staff and clinic resources) and the staff members responsible for screening, issuing referrals, and performing treatment for VTDR. This article focuses on workarounds to complete the latter from the perspectives of clinic staff involved in the process of preventing VTDR.

Introducing telemedicine interventions into a clinic can often lead to additional duties for medical staff, including staff ‘safety net’ primary care clinics. The need for collaboration across novel technologies, training, resource availability, staff motivation, and advice and guidance as a prerequisite for the implementation of telemedicine platforms is crucial [13, 14]. Currently, clinics can broadly approach telemedicine challenges in two ways: the ‘squeeze approach’ where staff are left to fit in the extra tasks, or the ‘redesign approach,’ in which existing and new work processes are restructured to accommodate new tasks and responsibilities. As this approach has not yet become commonplace, the ‘squeeze approach’ often becomes a necessity. These additional requirements tasked to medical staff (through the ‘squeeze approach’) engender the need for innovative practices, or workarounds, to ensure that screenings and appropriate follow-through occur [15–17].

Workarounds are attempts to fix a problem, that is, working in ways for which a technology, power structure, or knowledge system are not designed. They are part of the “informal temporary practices for handling exceptions to normal workflow” [18]. Several decades of work by sociologists of science and technology have examined how technology is used for purposes other than those for which it was designed, and to understand how the networks [19], logics [20, 21], and representational practices [22] enable and help form contextually shaped workarounds. Workarounds allow actors to use their discretion or resistance to the systems that constrain their work [19] and to fill and expand their responsibilities in order to meet the demands of care [23].

In terms of health services delivery, workarounds are most often discussed under the aegis of medical error or quality monitoring [24, 25]. They are increasingly recognized as both common and under-valued work by clinic staff in response to the increasing demands and complexity of health care delivery, although they can also be detrimental to patient safety [26, 27]. Workarounds drive the need to link individual practices to larger health care systems [13, 27], and to evaluate how novel workflows in contexts of dwindling resources can introduce novel risks to patient care [28, 29]. Furthermore, workarounds need to be understood in relation to facilitators and barriers created by local, state, and federal policies and regulations [30].

In this article, we suggest that workarounds can be performed to complete necessary tasks, but can also appear as “benevolent dissonance,” an overstepping of boundaries and disruption of proscribed workflows, not to be self-serving, but rather to provide care for this population with complex medical needs. Below, we present a glimpse into how the increased use of information technology and incorporation of specialty screening mechanisms into primary care is contingent upon workarounds performed by frontline clinic staff.

## Methods

All aspects of this study received Institutional Review Board approval. We identified all ‘safety-net’ clinics in California that have implemented EyePACS diabetic retinopathy screening program for at least 3 years and stratified the results based on geographic category (urban, rural, or suburban). Of the 112 total clinics identified, we contacted six randomly selected clinics by phone and site visits; the first from each geographical category that agreed to participate included two Federally Qualified Health Centers (FQHC) and one Federally Qualified Health Center-look-alike (as characterized by the U.S. Health Resources and Services Administration). This selection process allowed for sufficient variation across geographic and demographic contexts of FQHCs, which in turn enabled our exploration of common factors in the implementation of telemedicine screening for diabetic retinopathy. Pseudonyms are used for the clinic names: Charity Medical Center (urban clinic), Creekview County Medical Center (suburban clinic), and Summit Family Health Clinic (rural clinic). Both Charity and Creekview County have co-located family health/primary care as well as ophthalmology departments; Summit only provides family health, and patients must seek specialty care at outside clinics (none of which are located in the same town). A total of 1 week was spent at each location throughout December 2015–January 2016.

Three types of study participants were included at each site, including staff members, diabetic patients screened for diabetic retinopathy, and diabetic patients flagged for follow-up for VTDR. The latter two patient populations are described elsewhere [12]. A purposive sample of the range of key staff members affiliated with DR screening and treatment, including diabetes care management and education and non-clinical staff involved in the program implementation and management, were selected to participate in an interview and to be observed; no exclusion criteria were applied. Some staff members take on multiple roles across the diabetes screening and treatment workflow; for instance, a medical assistant could serve as a retinal imaging specialist, a scheduler, as well as a traditional allied health professional. Staff members received a token compensation for their participation.

An interview protocol was developed collaboratively by the study team (Additional file 1), drawing on existing literature on health services delivery, telemedicine, and diabetes management. The protocol ascertained information on the following topics: a staff member’s background, motivation for their work, clinic and cross-clinic communication, constraints staff face in their work, their role in and perceived effectiveness of the screening, referral, and treatment process, and perceptions of facilitators and barriers to preventing and/or treating VTDR. It also asked staff to describe the process of screening patients from start to finish. In addition, study participants were encouraged to elaborate on particular topics they deemed to be highly salient to the process, such as clinic technology (e.g. the electronic health record system) or additional issues related to care for adverse consequences of diabetes.

All interviews were audio-recorded and transcribed. Written notes from 100 h of participant observation performed by one author (Bouskill) were recorded. Interview transcriptions and notes were uploaded into the team-based, qualitative data analysis software program Dedoose [31]. A codebook, drawing on existing literature and pilot interviews, was developed based on the domains covered within the interview protocol. Coding was performed by two authors with extensive experience in qualitative data analysis (Bouskill and Smith-Morris). Coding occurred in two steps: two interviews were selected and independently coded. Any discrepancies were discussed and resolved until consensus was reached. Themes were identified inductively and deductively through recurrence, use of metaphor within narrative descriptions, and exceptional cases [32–34]. Descriptions of the screening process as it was described by staff were displayed in the aggregate. Lastly, participant observation notes were coded for instances of observed workarounds and discussions that followed them with staff.

The themes were identified within the various aforementioned domains covered within the interview protocol (e.g., constraints, staff role in the diabetes management process). The findings presented here center on constraints (or, “pain points”) and workarounds throughout the DR screening process stratified by medical staff (i.e. medical assistants) and clinical specialists (i.e. ophthalmologists).

## Results

A total of 23 staff members involved in the screening and treatment process for diabetic retinopathy participated in an interview. At Charity Medical Center, 8 staff members participated. Seven staff members from Summit Family Health Clinic and 8 from Creekview County Medical Center also took part in the study. The types and numbers of staff members who participated across all three clinics are depicted in Table 1.

**Table 1:** Study participants by type of staff

Type of Staff	Number of Participants (*N* = 23)
Medical Assistant^a^	8 (35%)
Appointment Scheduler	1 (4%)
Physician Assistant	1 (4%)
Primary Care Physician	2 (9%)
Optometrist	3 (13%)
Ophthalmologist	3 (13%)
Diabetes Educator	2 (9%)
Diabetes Health Coach	1 (4%)
Non-clinical Primary Care Manager	2 (9%)

^a^Note that Medical Assistants often served multiple roles, such as appointment schedulers

**Fig. 1:**
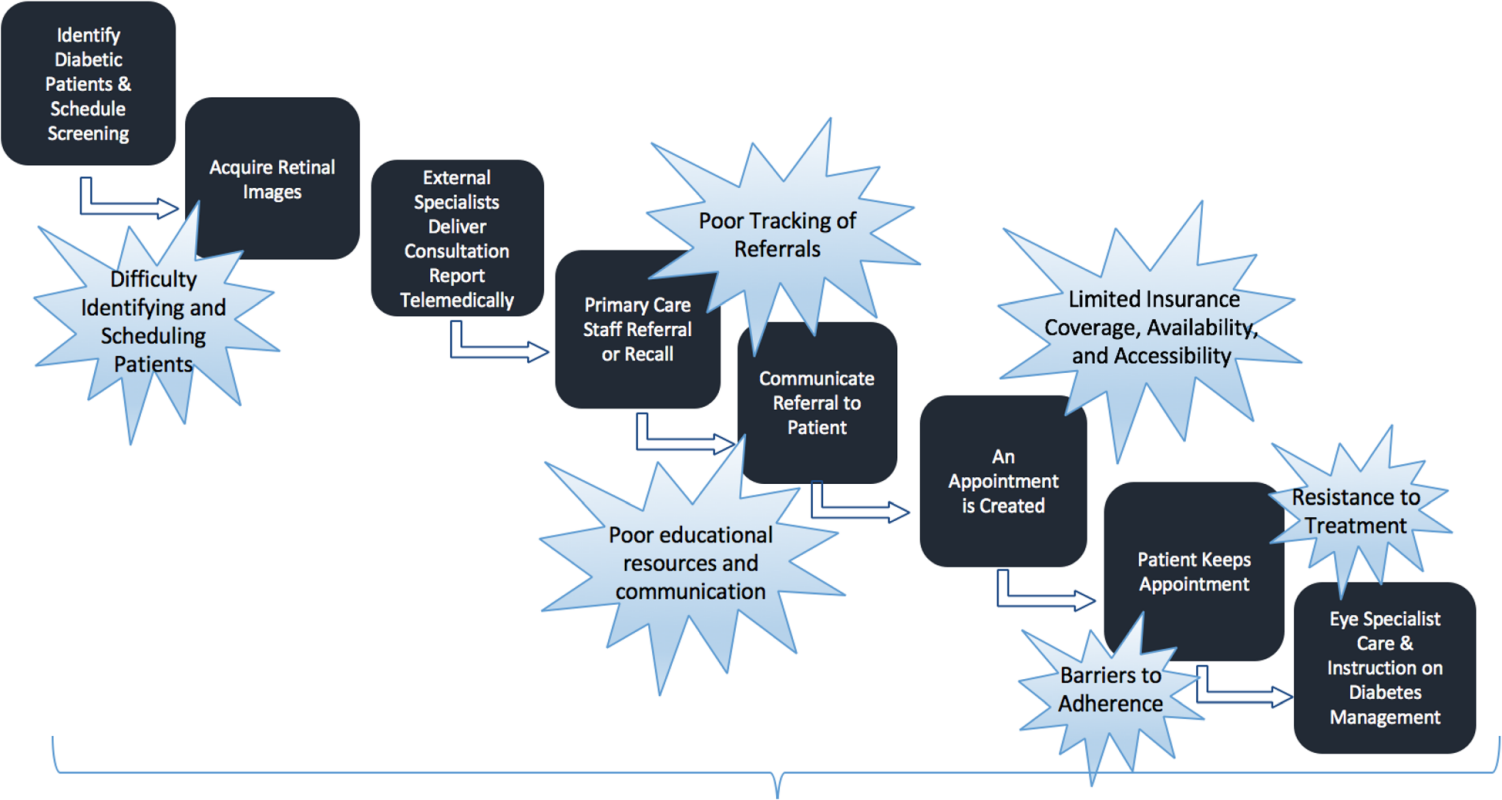
General Workflow and Associated Barriers to Diabetic Eye Care Screening and Treatment

Figure 1 outlines the standard workflow of DR screening through specialist treatment, overlaid with several pain points in the patient screening-referral-treatment workflow. These ranged from difficulty securing and performing screening appointments within primary care settings for patients with diabetes, scheduling patients for follow-up diagnostics/care, poor adherence to injections and/or laser treatment for VTDR, and poor management of diabetes. Observed and reported workarounds clustered around two types of staff members at opposite ends of the workflow and, interestingly, the clinic hierarchy: among medical assistants within family health/primary care settings and among ophthalmologists (e.g. retinal specialists). This is not to say that other staff member and clinicians do not perform workarounds; however, our sample revealed a frequency of workarounds described among medical assistants and ophthalmologists, which will be the focus of the findings. Although varied in type and agency, the workarounds described in the following highlight the persistent gaps telemedicine programs seek to fill as well as persistent issues, such as poor diabetes management or lack of insurance coverage, that are not rectified by telemedicine alone. Interestingly, the only difference observed across geographic regions were particular challenges to follow-up treatment in the rural clinic, as access to ophthalmological care was limited and required patients to travel longer distances; the urban and suburban clinics had in-house ophthalmology clinics.

It is also important to note that these workarounds in regard to screening and treatment for diabetic retinopathy occur above and beyond existing workarounds not related to DR screening within the resource-strapped and overpopulated clinics depicted here. For instance, one diabetes educator made due with a limited budget for educational materials by acting as a ‘Robin Hood’ and photocopying educational materials from other clinics with more resources. In the following, we outline how telemedical platforms and policies within primary care and across primary care and ophthalmological care, are shifting traditional roles without providing the necessary resources and communication to function effectively.

In accordance with the traditional definition of workarounds [18], we observed medical assistants consistently performing deviations from their outlined workflows. Key themes in regard to workarounds included means to identify patients needing to be screened, caution patients who will likely need follow-up care, and help move patients through the referral process to specialty care. With that, it would be remiss not to mention the personal motivation and expressions of camaraderie we observed across primary care staff. Medical assistants repeatedly reiterated feeling a deep connection to the patients and community, often volunteering in community events and sharing personal stories of family members affected by diabetes. All but one medical assistant was fluent in Spanish, which facilitated engagement with the largely Spanish-speaking population. Such emotional connections can act as a form of motivation in a context of increasing demands and constant resource depletion, and where the work of medical assistants can be taken-for-granted within the crowded and complex clinical space of primary care.

At the most basic level, medical assistants are responsible for supporting primary care physicians, physician assistants, nurses, and diabetes educators by performing routine tasks, such as moving patients around a clinic, taking vitals, and scheduling appointments. Medical assistants play a critical role in diabetic retinopathy screening, in addition to the multitude of additional tasks they must perform. Medical assistants have the important tasks of identifying, recruiting, scheduling, capturing, and uploading the retinal images. While medical assistants receive training on DR screening, they perform the exams without the guidance of the primary care physician, physician assistant, or nurse.

Clinical staff who manage medical assistants can either facilitate or hinder the work of medical assistants at various points along the process, including the earliest stage in the process– identifying and recruiting patients to be screened. The motivation to recruit is not only directly to benefit the individual patients, but also indirectly to maintain telemedicine programs like EyePACS at a prorated cost, i.e., clinics must meet certain quotas of patients screened per month, a task which is delegated to medical assistants. Although electronic health record systems should hasten and streamline the identification of diabetic patients needing screening, clinicians across clinics were unreliable in uploading the relevant data, such as a diabetes code, into the electronic medical record. Thus, when one medical assistant at Summit pulled patient records for recruitment, he realized that the number of patients was lower than a community-based estimate of patients with diabetes. At a closer glance at the electronic health records, the medical assistant noticed that patients with consistently high Hemoglobin-A1C levels (a hallmark of poorly-managed diabetes) were not coded as having diabetes in the system. When the medical assistant tried to send alerts to clinicians, they consistently landed in the clinicians’ spam filters or yielded no response. Unable to suggest changing to another system or to alert supervisors to the oversight, the medical assistant devised his own method of pulling patients with high Hemoglobin-A1C levels as a proxy for diabetes, and created a more extensive, accurate list of patients in need of diabetic retinopathy screening.

Another opportunity to screen patients comes when primary care physicians give a warm hand-off to medical assistant-photographers after seeing patients for a scheduled primary care physician appointment. Although medical assistants often compile lists in advance of incoming patients with diabetes, it is the work of clinicians to close the gap and inform patients that they should be screened at the close of a check-up. Yet, across clinics we observed medical assistants quite literally chasing after patients who were walking out of the clinic after an appointment without having been told by their physician that they should be screened.

These examples demonstrate informational technology shortcomings and oversights from clinicians that created complications downstream at the first critical vulnerability within the workflow– identifying and recruiting patients to be screened. Whereas it is the duty of medical assistants to screen the patients, it is likewise the domain of the primary care physicians to promote screening for diabetic retinopathy and to ensure that patients are transferred to the medical assistant for screening. While capturing patients before they leave clinic grounds was often a successful means of getting patients to attend screening, it was also disruptive, time-consuming, and potentially off-putting to patients. At all three clinics, medical assistants noted that they would prefer *not* having to perform patient outreach under such haphazard means, and that they would have benefitted from more systematic cooperation of primary care physicians in this phase of the process.

Medical assistants also face the particular quandary of lacking the qualifications to make diagnoses, but had often gained enough experience to recognize immediately upon capturing an image when a particular case will require follow-up ophthalmological care. Even though medical assistants may be fairly certain that a patient likely has VTDR based on the photographs they have taken, they are hamstrung in their ability to convey this to patients. Despite this, medical assistants described a workaround in which they would acknowledge not having a medical degree, but would ‘hint’ at the fact that patients will *really* need to follow-up, stating explicitly on one occasion at Charity, “Hey, you know, it looks like you might need further help… it’s up to us to even hint at it.” Another medical assistant noted, “I definitely know in some cases that it’s probably problematic. I’ll let the patients know that they’ll more than likely be contacted for follow-up, but it doesn’t mean that there is anything urgent going on, it’s just the doctor may want to take a closer look.” Medical assistants at all sites clearly overstepped their qualifications and duties, but did so to ensure that patients in dire need of ophthalmological care would adhere to follow-up treatment recommendations.

Medical assistants also recognized that patients in need of follow-up care may encounter barriers in scheduling follow-up care, an issue which was reported at all three clinics. At Summit, patients’ insurance coverage limited their options of finding available specialty care that was close to the local community; however, at Charity and Creekview, where ophthalmological care was available in-house, the central issue was a lack of available appointments. Here, medical assistants reported literally “walking down the urgent cases” to the ophthalmology appointment schedulers and urging them to take patients sooner than they otherwise would be willing to. As one medical assistant at Creekview explained, “I don’t want a patient to go blind, so I take that extra step and go to the scheduler in Ophthalmology for them. Not everybody does that though. Sometimes they will say their first available appointment is in one month, so I try to do my part so they can get in there as soon as they can.” This demonstrates another example of the lengths some medical assistants are willing to go to prevent blindness, as well as the potential for disparities for patients whose medical assistants may not perform the same workaround.

We observed compelling examples of workarounds at the opposite end of the program, that is, among ophthalmologists who are facing an influx of patients referred from the primary care DR screening program. The themes of workarounds among ophthalmologists included inclusion of diabetes management into ophthalmological care, addressing scheduling issues, and sharing retinal images that may not be approved for release.

As was the case among medical assistants (at the lower end of the medical personnel hierarchy), some workarounds observed among eye care clinicians (at the upper end of the medical personnel hierarchy) were performed out of sheer pragmatism, goodwill, or for mutually beneficial reasons, as was the case when an ophthalmologist described enrolling patients from the FQHC into clinical trials conducted at his private clinic to ensure that they would receive state-of-the-art care and extra attention, often not able to be provided at an FQHC.

At the outset, we must note that primary care physicians are the gatekeepers to the ophthalmologists by initiating the referral (none of the patients were part of a preferred-provider organization). With that, patients arrive in ophthalmological care primarily if a primary care physician has followed-up on the EyePACS recommendation to refer to specialty care. In dealing with the volume of patients scheduled into ophthalmology clinics, and the high number of ‘no-shows’ into the clinic, one ophthalmologist at Creekview admitted to tracking ‘repeat offenders’ (those who were often scheduled, but never came to their appointments) and scheduling one or two back-up appointments for the same time.

Be it good medicine or a good workaround, ophthalmologists were often observed taking time and energy to promote good management of diabetes and the utilization of primary care and endocrinology to do so. In two different clinics, two ophthalmologists even refused to see patients who did not have a primary care physician. While others did not turn away patients who are not routinely seen by primary care, they could understand the logic behind this workaround. In the same breath, these ophthalmologists at Creekview and Charity described how they actively educate patients on how diabetes management ties in directly with ophthalmological care; in other words, alerting patients to the fact that poor management of diabetes would counteract the care they received in ophthalmology. An ophthalmologist at Charity stated,

“When I pull up the chart note on here, I’m not just doing it to fill in my ‘eye’ part here. On the side here it shows medication they’re on and who their doctor is, so I can kind of go through this and see what, you know, how many medications the patient is on, not just for diabetes, but also for high blood pressure and other cardiovascular issues. And I can tell, this is somebody who is a sick diabetic and we need to make sure we do a thorough exam and they thoroughly understand what we’re trying to accomplish here.”

Another ophthalmologist at Creekview elaborated on this, stating it was the most important part of his role in ensuring patient adherence:

“I show up week in, week out, examining them, you know, going through their exam with them, asking them how they’re controlling their diabetes, what their primary care has told them from the last visit, specifically asking, “What was your last A1C level? Are you in the range you need to be? Do we really believe you’re under control? Do you think you’re under control?” You know, then I’ll give them the good news, hopefully, on the exam, and I’ll say, “Hey, it looks good. We don’t need to do laser.” [Interviewer: So, you’re really bringing in much more than just, just the eyes?] It really is. It’s not—like in my private practice, I have like maybe one patient per half-day in my private practice who’s, you know, a challenging patient. Here, I’m lucky if I have one patient who’s not a challenging patient.”

An additional interesting workaround employed by an ophthalmologist at Charity to increase adherence was the use of his own smart phone to share pictures of different retinal images. Done solely for the benefit of the patient, and without identifying information, this clinician stated, “Some patients, they really want to know what the heck is going on,” and that “[receiving treatment] is really a leap of faith” for many. He had taken photographs on his mobile phone of various retinal images, from healthy to severe cases, to show the progression of diabetic retinopathy. In his view, this was a workaround to account for lack of instructional materials in the clinic (of which none relating to DR screening and VTDR could be found), despite the fact that it is not generally advised to share patients’ images without their consent. Hence, the workarounds observed within specialty care help address the rising patient need for education, which is not readily supplied within primary care. This also points to the added benefit of including ophthalmologists in designing the communication strategies and materials on proper diabetes management.

The performance of workarounds by medical specialists at the top of the medical hierarchy demonstrates that these pragmatic, but irregular, adjustments are not solely performed by lower-ranked staff. Often, although not always, these workarounds were mutually beneficial to the ophthalmologist and patient. However, consistently, there was a recognized benefit and limited likelihood of cost when such workarounds took place.

## Discussion

In sum, we found that this telemedicine platform introduces new burdens on already-strapped ‘safety net’ clinics. In this example, these burdens included 1.) Primary care medical assistants must ensure that diabetic patients have retinal screening on schedule, and, in some case, that quotas are met in order to retain the telemedicine screening technology, and 2.) Specialty care clinics must account for the rising patient burden induced by increased access to screening and coupled with limited adherence to complicated treatment protocols. While telemedicine offers needed resources, the presence of additional screening tools places significant burdens on existing care resources. In our research, telemedical screening, including both the novel technology and policies behind its use, is shifting traditional roles within and across clinics.

These findings echo the growing body of work exploring workarounds within health care settings, as well as the tensions among workarounds as a threat to patient safety and as a means of overcoming systemic shortcomings and resource constraints [28, 29, 35]. For example, a recent study of workarounds in hospital electronic prescribing systems found workarounds as a result of time pressures induced by learning new technological systems as well as increasing demands and duties tasked to clinic staff [28].

Ultimately, the workarounds in this telemedicine program appear in part to be indicative of underlying problems in inter- and intra-clinic communication and disruptions in role clarity observed within models of health care delivery [36]. Not surprisingly, these stoppages and barriers are compounded by the complexity of diabetes management within resource-poor environments, both within and outside of the clinic [37–39]. They also point to the need for upfront planning prior to the introduction of telemedicine interventions in order to understand existing issues and reduce downstream complexity, as was found in a recent evaluation of a US-based telemedicine initiative [40].

The absence of workflow adjustments and lack of resources to accommodate scheduling and follow-up treatment have opened up space for the existence of workarounds, or improvised solutions to workflow strain. These workarounds are evidence of the fact that the success of telemedicine is a tenuous one, premised at times on the goodwill, benevolent dissonance, or sheer frustration of clinical staff [12, 41].

An additional issue that has not been systematically addressed are the ethical implications of improved access to screening for conditions such as diabetic retinopathy without ensuring equitable access to treatment. Although the reasons for lack of follow-up and treatment adherence are multifactorial (e.g., lack of access to care, fear of treatment, poor management of diabetes), in many cases, treatment barriers caused by inefficient appointment systems or lack of nearby, in-network clinics exacerbated an already complex pathway of preventing permanent vision loss. For patients with uncontrolled diabetes, a single screening is just one of numerous medical tasks to fulfill. In this state of chronic, multi-morbidity care, staff have an added role in helping patients respond to retinal screening results.

Medical assistants are many, have high turnover rates, and are often unsupervised during the recruitment and screening process. Ophthalmologists are rarely questioned or contested by other clinic staff (with the exception of a department head or hospital director). Thus, staff responsibilities for navigating patients through the complexities of diabetes management can be unclear or tenuous.

Ideally, the implementation of telemedical screening platforms would begin with a participatory, iterative, co-design framework that takes into account staff needs and functions at the front line in order to understand and optimize new workflows. Before this is commonplace, the presence of workarounds in order to meet expected duties and tasks, should be given close attention– not as deviations from standard workflows to be corrected, but as an initial step towards adapting to changes within complex health care delivery systems and creating cross-influences among expectations of administrators, the constraints of frontline staff, and the complexities of living with chronic disease(s) and their complications. However, this work can be time-consuming up front and potentially be seen as burdensome to staff who must both participate in the co-design, as well as allow for the presence of researchers in the clinic. In addition, clinic staff may also be reluctant to admit to their use of workarounds and in some cases, may not realize that their practices are in fact deviations from clinic workflows. Disentangling these dynamics can enhance the success of future telemedicine interventions.

## Conclusions

Despite noteworthy progress in detecting diabetic retinopathy and access to DR screening, telemedical screening adds additional burdens to medical staff that should be addressed to strengthen the potential of such platforms. The additional needs identified by new screening processes, when not met through additional follow-up resources, leaves frontline staff in the uncomfortable position of having to witness inequality and resource constraints without the ability to systematically address them, leading them to conduct irregularities in workflow, i.e. workarounds.

Given the burgeoning population of diabetic patients across the United States and the globe, shedding light on workarounds can be an important first step in identifying vulnerabilities and gaps in the recruitment-screening-treatment process. Furthermore, an in-depth analysis of workarounds can ultimately lead to the improved implementation of telemedicine where it is critically needed.

## Additional file

Additional file 1: Interview Guide. This guide was used when performing interviews across the range of clinicians and clinic/administrative staff members. (DOCX 34 kb)

## Abbreviations

DR screening: Diabetic retinopathy screening
VTDR: Vision-threatening diabetic retinopathy

## References

[1] Ward MM, Jaana M, Natafgi N (2015). Systematic review of telemedicine applications in emergency rooms. Int J Med Inform.

[2] Eedy DJ, Wootton R (2001). Teledermatology: a review. Br J Dermatol.

[3] Monnier J, Knapp RG, Frueh BC (2003). Recent advances in telepsychiatry: an updated review. Psychiatr Serv.

[4] Armfield NR, Coulthard MG, Slater A, McEniery J, Elcock M, Ware RS, Scuffham PA, Bensink ME, Smith AC (2014). The effectiveness of telemedicine for paediatric retrieval consultations: rationale and study design for a pragmatic multicentre randomised controlled trial. BMC Health Serv Res.

[5] Aiello LP, DCCT/EDIC research group (2014). Diabetic retinopathy and other ocular findings in the diabetes control and complications trial/epidemiology of diabetes interventions and complications study. Diabetes Care.

[6] Fong DS, Aiello L, Gardner TW, King GL, Blankenship G, Cavallerano JD, Ferris FL, Klein R, American Diabetes A (2004). Retinopathy in diabetes. Diabetes Care.

[7] Klein BE (2007). Overview of epidemiologic studies of diabetic retinopathy. Ophthalmic Epidemiol.

[8] Kirkizlar E, Serban N, Sisson JA, Swann JL, Barnes CS, Williams MD (2013). Evaluation of telemedicine for screening of diabetic retinopathy in the veterans health administration. Ophthalmology.

[9] Taylor CR, Merin LM, Salunga AM, Hepworth JT, Crutcher TD, O’Day DM, Pilon BA (2007). Improving diabetic retinopathy screening ratios using telemedicine-based digital retinal imaging technology: the Vine Hill study. Diabetes Care.

[10] Cuadros J, Bresnick G (2009). EyePACS: an adaptable telemedicine system for diabetic retinopathy screening. J Diabetes Sci Technol.

[11] Quade R (2011). Evaluation of the expanding access to diabetic retinopathy screening initiative. Evaluation report, California HealthCare Foundation.

[12] Smith-Morris, C, Bresnick, GH, Cuadros, J, Bouskill, KE, Pedersen, ER. Diabetic Retinopathy and the Cascade into Vision Loss. Med Anthropol. 2018; 10.1080/01459740.2018.1425839. 10.1080/01459740.2018.1425839 29338335

[13] Sobo EJ (2016). Culture and meaning in health services research: an applied approach.

[14] Sørensen T, Dyb K, Rygh E, Salvesen R, Thomassen L (2014). A qualitative description of telemedicine for acute stroke care in Norway: technology is not the issue. BMC Health Serv Res.

[15] Ferneley EH, Sobreperez P (2006). Resist, comply or workaround? An examination of different facets of user engagement with information systems. Eur J Inf Syst.

[16] Halbesleben JRB, Wakefield DS, Wakefield BJ (2008). Work-arounds in health care settings: literature review and research agenda. Health Care Manag Rev.

[17] Koppel R, Wetterneck T, Telles JL, Karsh B-T (2008). Workarounds to barcode medication administration systems: their occurrences, causes, and threats to patient safety. J Am Med Inform Assoc.

[18] Kobayashi M, Fussell SR, Xiao Y, Seagull FJ (2005). Work coordination, workflow, and workarounds in a medical context. CHI'05 extended abstracts on Human factors in computing systems: 2005.

[19] Pollock N (2005). When is a work-around? Conflict and negotiation in computer systems development. Sci Technol Hum Values.

[20] Berg M, Mol A (1998). Differences in medicine: unraveling practices, techniques, and bodies.

[21] Berg M, Timmermans S (2000). Orders and their others: on the constitution of universalities in medical work. Configurations.

[22] Suchman L (1995). Making work visible. Commun ACM.

[23] Timmermans S, Freidin B (2007). Caretaking as articulation work: the effects of taking up responsibility for a child with asthma on labor force participation. Soc Sci Med.

[24] Spear SJ, Schmidhofer M (2005). Ambiguity and workarounds as contributors to medical error. Ann Intern Med.

[25] Vogelsmeier AA, Halbesleben JR, Scott-Cawiezell JR (2008). Technology implementation and workarounds in the nursing home. J Am Med Inform Assoc.

[26] Debono DS, Greenfield D, Travaglia JF, Long JC, Black D, Johnson J, Braithwaite J (2013). Nurses' workarounds in acute healthcare settings: a scoping review. BMC Health Serv Res.

[27] Johnson JK, Miller SH, Horowitz SD, Henriksen K, Battles JB, Keyes MA (2008). Systems-Based Practice: Improving the Safety and Quality of Patient Care by Recognizing and Improving the Systems in Which We Work. Advances in Patient Safety: New Directions and Alternative Approaches (Vol 2: Culture and Redesign).

[28] Cresswell KM, Mozaffar H, Lee L, Williams R, Sheikh A (2017). Workarounds to hospital electronic prescribing systems: a qualitative study in English hospitals. BMJ Quality &amp;amp; Safety.

[29] Ser G, Robertson A, Sheikh A (2014). A qualitative exploration of workarounds related to the implementation of national electronic health records in early adopter mental health hospitals. PLoS One.

[30] Buchbinder M. Comments delivered at the American Anthropological Association annual meetings. In: American Anthropological Association annual meetings: November 20, 2017 2016. Minneapolis: American Anthropological Association; 2016.

[31] SocioCultural Research Consultants, LLC (2016). Dedoose Version 7.0.23.

[32] LeCompte MD, Schensul JJ (1999). Designing and conducting ethnographic research.

[33] Ryan GW, Bernard HR (2003). Techniques to identify themes. Field methods.

[34] Strauss A, Corbin J, Norman KD, Vannaeds SLY (1994). Grounded Theory Methodology—An Overview. Handbook of Qualitative Research.

[35] van der Veen W, van den Bemt PMLA, Wouters H, Bates DW, Twisk JWR, de Gier JJ, Taxis K, Group BS, Duyvendak M, Luttikhuis KO (2017). Association between workarounds and medication administration errors in bar-code-assisted medication administration in hospitals. J Am Med Inform Assoc.

[36] Daykin N, Clarke B (2000). ‘They’ll still get the bodily care’. Discourses of care and relationships between nurses and health care assistants in the NHS. Sociol Health Illn.

[37] Smith-Morris C (2008). Diabetes among the pima: stories of survival.

[38] Smith-Morris C, Manderson L, Smith-Morris C (2010). The chronicity of life, the acuteness of diagnosis. Chronic conditions, fluid states: Chronicity and the anthropology of illness.

[39] Weaver LJ, Mendenhall E (2014). Applying syndemics and chronicity: interpretations from studies of poverty, depression, and diabetes. Med Anthropol.

[40] Stevenson L, Ball S, Haverhals LM, Aron DC, Lowery J (2016). Evaluation of a national telemedicine initiative in the veterans health administration: factors associated with successful implementation. J Telemed Telecare.

[41] Solimeo SL, Ono SS, Lampman MAM, Paez MBW, Stewart GL (2015). The empowerment paradox as a central challenge to patient centered medical home implementation in the Veteran's health administration. J Interprof Care.

