# Supplementary material for: Blind spots in telemedicine: a qualitative study of staff workarounds to resolve gaps in diabetes management
Source: BMC Health Serv Res. 2018 Aug 7;18:617. doi: 10.1186/s12913-018-3427-9 (PMC6081904; doi:10.1186/s12913-018-3427-9)
Supplement: Supplementary file 1 — Interview Guide. This guide was used when performing interviews across the range of clinicians and clinic/administrative staff members. (DOCX 34 kb) [file 12913_2018_3427_MOESM1_ESM.docx]

## Interviews with Clinicians/Staff Study ID # ________

**Demographics:**

Age: _______ Gender: ______ Birthplace: ________________ Languages: __________

Training/Degree/Cert: _______________ Profession/Title: _______________________

1. Please describe and explain the existing procedures/actual activities in place to:
   1. Prescribe screening (or walk-in)
   2. Track patient attendance at screening appointment
   3. Reach out to patient in for (repeat) screening
   4. Register patient at arrival
   5. Capture fundus images
      1. What is said? How is it said?
      2. What are your tricks for capturing a good image? Do you feel you’re particularly good at this?
   6. Prepare fundus images for [remote] reading
   7. Receive readings/results, reports and recommendations
   8. Evaluate/annotate readings/results
   9. Distribute readings/results to PCP/staff
   10. Make referral, accompanied by readings/results to optometrist
   11. Make referral, accompanied by readings/results to ophthalmologist
   12. Inform patient of readings/results
   13. Schedule patient for discussion of readings/results
   14. Track patient attendance at referral appointment to optometrist and/or ophthalmologist
   15. Receive/track report from referral appointment to optometrist and/or ophthalmologist
2. Please comment on any usual discrepancy between (semi) formal procedure and actual work.
   1. Are there any “workarounds” that you use to do your work better?
   2. If a problem is identified, can staff resolve it? Who is a good person to go to when you have a problem that needs solving?
3. [Specifically for medical assistants who serve as photographers] Looking at required technical skills and quality of image capture:
   1. What is your training background?
   2. Have you worked with other kinds of fundus cameras?
   3. How do you assess if a photo has proper quality?
   4. What do you find particularly difficult or challenging doing retinal photography (think of the entire process from going to get the patient, through the screening, to handling of the photos for post processing)?
4. What do you find particularly interesting and/or rewarding about your work?
   1. Do you talk with patient about eye problems? About diabetes and care? About follow up appointments?
5. What do you believe are the variables that contribute to patient adherence to recommendations?
   1. Patient attitude, beliefs, resources, knowledge
   2. Staff attitude, beliefs, resources, knowledge
   3. Systemic issues, such as wait times, lack of time to explain, lack of regular check-in?
6. Have any patients surprised you in their compliance? What “types” of patients do you feel are most likely to be compliant/non-compliant?
7. What do you think is the most important part of what you do to affect compliance?
8. Have you run across patients that feel eye problems are inevitable?
9. If a problem is identified, who’s responsibility is it to take the health care to the next level? Who has responsibilities in making that happen? (looking here for staff sense of responsibility vs. sense that “if the patient doesn’t want to get better, it’s their choice”)
